# Gut microbiota and risk of iron deficiency anemia: A two-sample Mendelian randomization study

**DOI:** 10.1097/MD.0000000000041617

**Published:** 2025-02-21

**Authors:** Wenhui Lei, Zhaoyun Liu, Hai-Ping Lai, Rong Fu

**Affiliations:** a Department of Hematology, Tianjin Medical University General Hospital, Tianjin, PR China; b Tianjin Institute of Hematology, Tianjin, China; c Department of Internal Medicine, Lishui Municipal Central Hospital, Lishui, Zhejiang Province, PR China; d Tianjin Key Laboratory of Bone Marrow Failure and Malignant Hemopoietic Clone Control, Tianjin, China; e Department of Medicine, Ganzhou Tumor Hospital, Ganzhou, Jiangxi Province, PR China.

**Keywords:** genome-wide association studies, gut microbiota, iron-deficiency anemia, Mendelian randomization

## Abstract

Previous studies have suggested a link between gut microbiota and iron-deficiency anemia (IDA). However, interpreting these findings is difficult due to various factors that influence microbiome composition and the limitations of observational studies, such as confounding variables and reverse causation. This study aims to explore the causal relationship between gut microbiota and IDA using Mendelian randomization (MR) to overcome these limitations. We conducted a 2-sample MR analysis using data from genome-wide association studies from the MiBioGen Consortium and the UK Biobank. The gut microbiome data included 211 genus-level microbes linked to single-nucleotide polymorphisms from 18,340 participants in the MiBioGen Consortium. The outcome data for IDA were obtained from 484,598 participants in the UK Biobank, with 2941 cases and 481,657 controls. We assessed causal relationships using various MR techniques, primarily inverse variance weighting, and performed sensitivity analyses to confirm the robustness of our results. Nine genus-level gut microbes were significantly associated with IDA (*P* < .05). Protective factors included Clostridia, Actinomycetaceae, Pasteurellaceae, Oscillospira, Prevotella, and Roseburia, while risk factors included *Ruminococcus gnavus* group, Hungatella, and Parasutterella. Sensitivity analyses showed the reliability of these findings without significant variability. This study provides evidence for a causal relationship between specific gut bacteria and IDA risk, identifying potential targets for therapies aimed at improving outcomes for those with IDA. Further research is needed to clarify the bacteria involved.

## 1. Introduction

Iron-deficiency anemia (IDA) is a prevalent hematologic disorder and the most common nutritional deficiency globally, affecting approximately 30% of the population.^[1]^ While IDA is primarily linked to gastrointestinal bleeding and menstrual loss in females, reduced dietary iron intake and impaired absorption are also significant contributors.^[2]^ It can adversely impact children’s growth and development and compromise immune function. Long-term consequences include cardiomegaly, myocardial hypertrophy, heart failure, mental fatigue, concentration difficulties, and memory impairments. Thus, IDA poses considerable risks to cardiovascular, digestive, and nervous systems, as well as overall productivity. Despite advancements in global health, prioritizing IDA remains crucial for clinical professionals due to its extensive implications.

Observational studies indicate a strong relationship between gut microbiota and IDA, as the microbiota can influence the absorption of dietary iron. A comparative study of gut microbiota in 10 IDA patients and 10 healthy infants showed significant differences, including an increase in Enterobacteriaceae and Veillonellaceae and a decrease in Lachnospiraceae among the IDA cohort.^[3]^ Additionally, iron supplementation with hydrolyzed peptides may enhance microbial diversity and alleviate dysbiosis associated with IDA.^[4]^ Animal studies suggest IDA induces dysbiosis primarily in the distal gastrointestinal tract, correlating with increased short-chain fatty acids (SCFAs). Furthermore, research indicates that fermented goat milk can improve gut microbiota and intestinal barrier function in IDA patients. Other studies have shown that iron supplements may increase diarrhea risk by altering gut microbiota composition, leading to reduced beneficial bacteria and increased pathogenic strains.^[5,6]^

Given the potential reverse causation and bias in observational studies, Mendelian randomization (MR) offers a method to explore causal relationships between gut microbiota and IDA.^[7]^ MR leverages genetic variations as instrumental variables (IVs) to estimate causal links between exposures and disease outcomes, minimizing confounding factors.^[8–14]^ In this study, a 2-sample MR analysis was conducted using summary statistics from whole-genome association studies (GWAS) data from MiBioGen and the UK Biobank to assess the causal relationship between gut microbiota and IDA.

## 2. Methods

### 2.1. Research design

**Figure 1.**
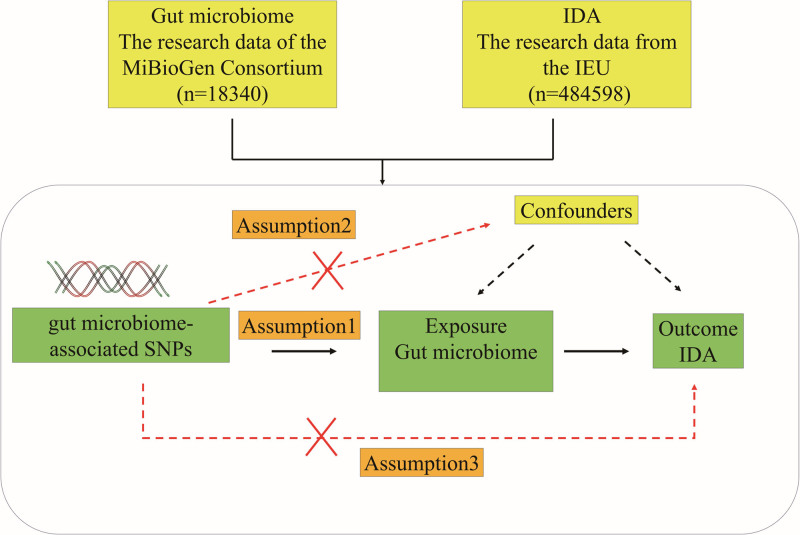
Research design framework: the study must adhere to the following conditions. Assumption 1: the genetic variations serving as instrumental variables must be significantly associated with the gut microbiome. Assumption 2: the genetic variations must be independent of any common and unmeasured confounders. Assumption 3: the SNPs must influence IDA solely through their association with the gut microbiome. IDA = iron-deficiency anemia, SNPs = single-nucleotide polymorphisms.

We employed a 2-sample MR study design to investigate the causal relationship between the gut microbiome and IDA, as outlined in Figure 1. To ensure the effectiveness of the IV in the MR design, 3 fundamental assumptions must be met: the genetic variations serving as IVs must be significantly associated with the gut microbiome (as the exposure); the genetic variations must be independent of any common and unknown confounders; the variations must only affect IDA through their relationship with the gut microbiome. The summary data primarily relied on independent genome-wide association studies (GWAS). MR utilized single-nucleotide polymorphisms (SNPs) to assess the causal relationship between the exposure of the gut microbiome and the outcome of IDA.

### 2.2. Data sources

The genetic variation data of the gut microbiome was sourced from the research data of the MiBioGen Consortium.^[15]^ This dataset investigated the whole-genome genotypes and fecal microbiome 16S rRNA gene data of 18,340 individuals from 24 cohorts, representing the largest meta-analysis of whole-genome data to date. The majority of the study population were of European descent (n = 13,266). The study focused on sequencing the microbial 16S rRNA gene variable regions V4, V3 to V4, and V1 to V2, and applied microbiome quantitative trait locus (mbQTL) mapping analysis to identify host genetic variations associated with genetic loci related to bacterial taxa abundance levels in the gut microbiome.^[15]^ Genus is the lowest taxonomic level, and a total of 211 genera with an average abundance exceeding 1% were identified, including 12 unknown genera.

The genetic data for GWAS of IDA was obtained from the IEU Open GWAS database, accessible at https://gwas.mrcieu.ac.uk/datasets,^[16]^ sourced from the UK Biobank data.^[16]^ The study cohort comprised 484,598 participants, with 2941 cases and 481,657 controls (gwas_id: ebi-a-GCST90038659). The summary-level data for IDA were derived from a GWAS study conducted by Handan Melike Dönertaş et al, involving 484,598 European participants and covering 9,587,836 SNPs.^[16]^

### 2.3. Instrumental variable selection

We employed a set of rigorous selection criteria to screen for eligible genetic IVs: Due to the limited number of IVs meeting the significance threshold (*P* < 5 × 10^−8^), we opted for a relatively less stringent threshold (*P* < 1 × 10^−5^) based on previous studies^[17–22]^ to capture genetic IVs that may be enriched for associations, thus obtaining more comprehensive results. We conducted a clumping procedure (*R*^2^ < 0.001, window size = 10,000 kb) to exclude SNPs in strong linkage disequilibrium states and ensure the independence of each SNP. SNPs with minor allele frequencies < 0.01, ambiguous SNPs with inconsistent alleles, and palindromic SNPs were excluded. Potential confounding factors^[23,24]^ such as hypermenorrhea and malnutrition were addressed by identifying and excluding SNPs associated with these factors using LDtrait Tool (https://ldlink.nih.gov/?tab=ldtrait). If a genetic IV was found to be associated with any other known phenotypes, it was excluded from subsequent MR analyses.

### 2.4. MR analysis

Before conducting MR analysis, the *F*-statistic of the microbiome IVs was calculated to assess the presence of weak IV bias, with an *F*-statistic < 10 indicating weak IV bias. The *F*-statistic is derived from the formula *F* = [(N − *K* − 1)/*K*] × [*R*^2^/(1 − *R*^2^)], where N represents the sample size of the exposure factor, *K* denotes the number of IVs, and *R*^2^ signifies the proportion of exposure factor variation explained by the IVs. In this study, 5 methods were employed to examine the causal relationship between the gut microbiome and IDA: Inverse variance weighted (IVW), simple model, MR-Egger, Weighted median, and weighted model techniques were utilized to evaluate the association between gut microbiota and IDA risk. The IVW method is considered the most accurate and powerful approach for estimating causal effects when all selected SNPs are valid IVs.

Odds ratios (ORs) were employed as the outcome measure, with IVW as the primary method. Standard inverse variance weighted analysis methods are susceptible to heterogeneity or pleiotropy influence. Therefore, sensitivity analyses were conducted to assess the validity and robustness of the IVW results. To address bias from horizontal pleiotropy, MR-PRESSO (MR pleiotropy RESidual sum and outlier) was utilized to detect potential horizontal pleiotropy. Additional tests for heterogeneity, including the MR-Egger intercept test, and corrected Cochran *Q*-statistic, were conducted to verify the reliability of statistically significant findings.

All statistical tests were 2-tailed, and due to the binary nature of the results, effect estimates were transformed into OR to elucidate the relationship between the gut microbiome and IDA. Results were considered statistically significant at a significance level of *P* < .05. Cochran *Q*-statistics were used to assess heterogeneity among SNPs, and a “leave-one-out” analysis was conducted to investigate the impact of individual SNPs on the causal association.

The statistical analyses were meticulously conducted utilizing the “TwoSampleMR” and “MR-PRESSO” packages within the R software framework, specifically version 4.3.3, ensuring rigor and precision in the data evaluation process.

## 3. Results

**Figure 2.**
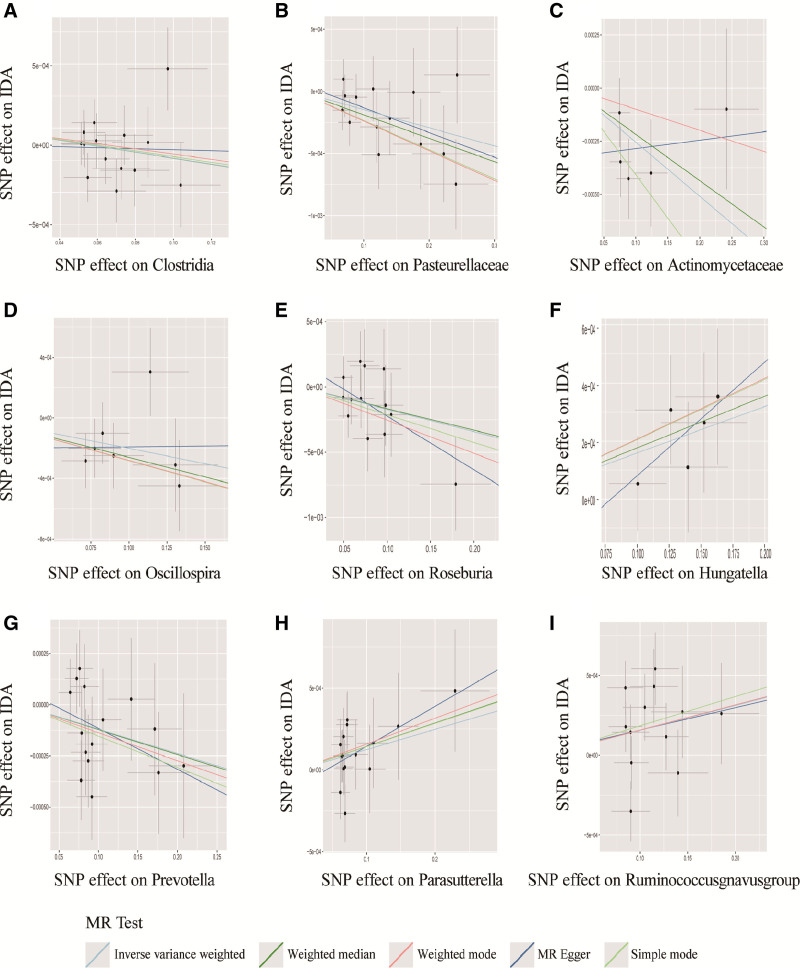
Scatter plots illustrating the causal effects of gut microbiota on IDA. Note: (A) Clostridia-IDA; (B) Pasteurellaceae-IDA; (C) Actinomycetaceae-IDA; (D) Oscillospira-IDA; (E) Roseburia-IDA; (F) Hungatella-IDA; (G) Prevotella-IDA; (H) Parasutterella-IDA; (I) *Ruminococcus gnavus* group-IDA. IDA = iron-deficiency anemia.

Based on the screening criteria, a total of 14,587 SNPs were identified as IVs for 211 genus-level gut microbes. All selected IVs demonstrated F statistics exceeding 10, suggesting a low likelihood of weak instrument bias. Through the Inverse Variance Weighting (IVW) method, 9 genus-level gut microbes were found to exhibit potential associations with IDA, as summarized in Table 1. The graphical representations in scatter plots illustrate the relationships between the effects of SNPs on these 9 genus-level gut microbes and their impact on IDA (Fig. 2). The estimated causal associations between these 9 genera and IDA are detailed in Table 1.

**Table 1:** Significant MR results of causal association between gut microbes and IDA.

Exposure	Method	n.snp	Beta	SE	*P*	95% CI	Horizontal pleiotropy *P* for Egger intercept	Heterogeneity (*P* for Cochran *Q*)	MR-PRESSO results *P* (global test)
class.Clostridia	MR-Egger	14	−0.0003	0.004	.92	(−0.009,0.008)	.65	.61	74
	Weighted median	14	−0.0023	0.001	.05	(−0.005,0.001)			
	Inverse variance weighted	14	−0.0023	0.001	8.37 × 10^−3^	(−0.004, −0.001)			
	Simple mode	14	−0.0022	0.002	.29	(−0.006,0.001)			
	Weighted mode	14	−0.0020	0.002	x.34	(−0.006,0.002)			
family.Actinomycetaceae	MR-Egger	5	0.0003	0.002	.87	(−0.004,0.004)	.25	.38	.43
	Weighted median	5	−0.0021	0.001	.05	(−0.004,0.000)			
	Inverse variance weighted	5	−0.0025	0.001	4.5 × 10^−3^	(−0.004, −0.001)			
	Simple mode	5	−0.0041	0.001	.09	(−0.008, −0.001)			
	Weighted mode	5	−0.0009	0.002	.59	(−0.004,0.002)			
family.Pasteurellaceae	MR-Egger	14	−0.0020	0.001	.14	(−0.004,0.000)	.65	.83	.62
	Weighted median	14	−0.0019	0.001	.01	(−0.003, −0.001）			
	Inverse variance weighted	14	−0.0015	0.001	7.27 × 10^−3^	（−0.002, −0.001)			
	Simple mode	14	−0.0023	0.001	.11	(−0.005,0.000)			
	Weighted mode	14	−0.0024	0.001	.09	(−0.005,0.000)			
genus.*Ruminococcus gnavus* group	MR-Egger	12	0.0014	0.003	.67	(−0.005,0.007	.96	.11	.14
	Weighted median	12	0.0015	0.001	.06	(−0.001,0.003)			
	Inverse variance weighted	12	0.0016	0.001	.02	(0.001,0.002)			
	Simple mode	12	0.0018	0.001	.18	(−0.000,0.004)			
	Weighted mode	12	0.0015	0.001	.21	(−0.001,0.003)			
genus.Hungatella	MR-Egger	5	0.0039	0.003	.39	(−0.003,0.011)	.60	.89	.90
	Weighted median	5	0.0018	0.001	.04	(0.000,0.003)			
	Inverse variance weighted	5	0.0016	0.001	.02	(0.001,0.002)			
	Simple mode	5	0.0021	0.001	.15	(−0.000,0.004)			
	Weighted mode	5	0.0020	0.001	.14	(−0.000,0.004)			
genus.Oscillospira	MR-Egger	7	0.0001	0.004	.97	(−0.007,0.008)	.61	.59	.33
	Weighted median	7	−0.002	0.001	.02	(−0.005, −0.001)			
	Inverse variance weighted	7	−0.002	0.001	.02	(−0.004, −0.001)			
	Simple mode	7	−0.0020	0.001	.14	(−0.006,0.001)			
	Weighted mode	7	−0.0020	0.001	.15	(−0.006,0.001)			
genus.Parasutterella	MR-Egger	15	0.0020	0.001	.21	(−0.001,0.006)	.51	.77	.88
	Weighted median	15	0.0014	0.001	.08	(−0.000,0.003)			
	Inverse variance weighted	15	0.0012	0.001	.04	(0.001,0.002)			
	Simple mode	15	0.0014	0.001	.30	(−0.001,0.004)			
	Weighted mode	15	0.0015	0.001	.25	(−0.001,0.004)			
genus.Prevotella	MR-Egger	15	−0.0020	0.001	.26	(−0.005,0.001)	.62	.63	.83
	Weighted median	15	−0.0012	0.001	.12	(−0.003,0.000)			
	Inverse variance weighted	15	−0.0011	0.001	.03	(−0.002, −0.001)			
	Simple mode	15	−0.0015	0.001	.26	(−0.004,0.001)			
	Weighted mode	15	−0.0013	0.001	.28	(−0.003,0.001)			
genus.Roseburia	MR-Egger	13	−0.0041	0.002	.11	(−0.009,0.000)	.31	.75	.85
	Weighted median	13	−0.0016	0.001	.15	(−0.004,0.000)			
	Inverse variance weighted	13	−0.0017	0.001	.04	(−0.003,0.00001)			
	Simple mode	13	−0.0021	0.001	.28	（−0.006,0.002）			
	Weighted mode	13	−0.0025	0.001	.22	（−0.006,0.001）			

As shown in Table 1 and the scatter plot (Fig. 2), 9 gut microbiota species including Clostridia, Actinomycetaceae, Pasteurellaceae, *Ruminococcus gnavus* group, Hungatella, Oscillospira, Parasutterella, Prevotella, and Roseburia were found to be associated with IDA through the MR method using the IVW approach (all *P* < .05). It is notable that Clostridia (IVW: beta = −0.0023, *P* = 8.37 × 10^−3^ 95% CI [−0.004 to −0.001]), Actinomycetaceae (IVW: beta = −0.0025,*P* = 4.5 × 10^−3^, 95% CI (−0.004, to −0.001), Pasteurellaceae (IVW: beta = −0.0015, *P* = 7.27 × 10^−3^, 95% CI [−0.002 to −0.001]), Oscillospira (IVW: beta = −0.0020, *P* = .02, 95% CI [−0.004 to −0.001]), Prevotella (IVW: beta = −0.0011, *P* = .03, 95% CI [−0.002 to −0.001]), and Roseburia (IVW: beta = −0.0017, *P* = .04, 95% CI [−0.003 to −0.0001]) appeared to be protective factors against IDA, while *R gnavus* group (IVW: beta = 0.0016, *P* = .02, 95% CI [0.001 to −0.002]), Parasutterella (IVW: beta = 0.0012, *P* = .04, 95% CI [0.001 to 0.0002]), and Hungatella (IVW: beta = 0.0016, *P* = .02, 95% CI [0.001–0.002]) were identified as risk factors for IDA. To further investigate the influence of IDA on gut microbiota, a reverse MR analysis revealed no shared IVs between IDA and these 9 gut bacteria, indicating that IDA does not affect these microbiota species.

**Figure 3.**
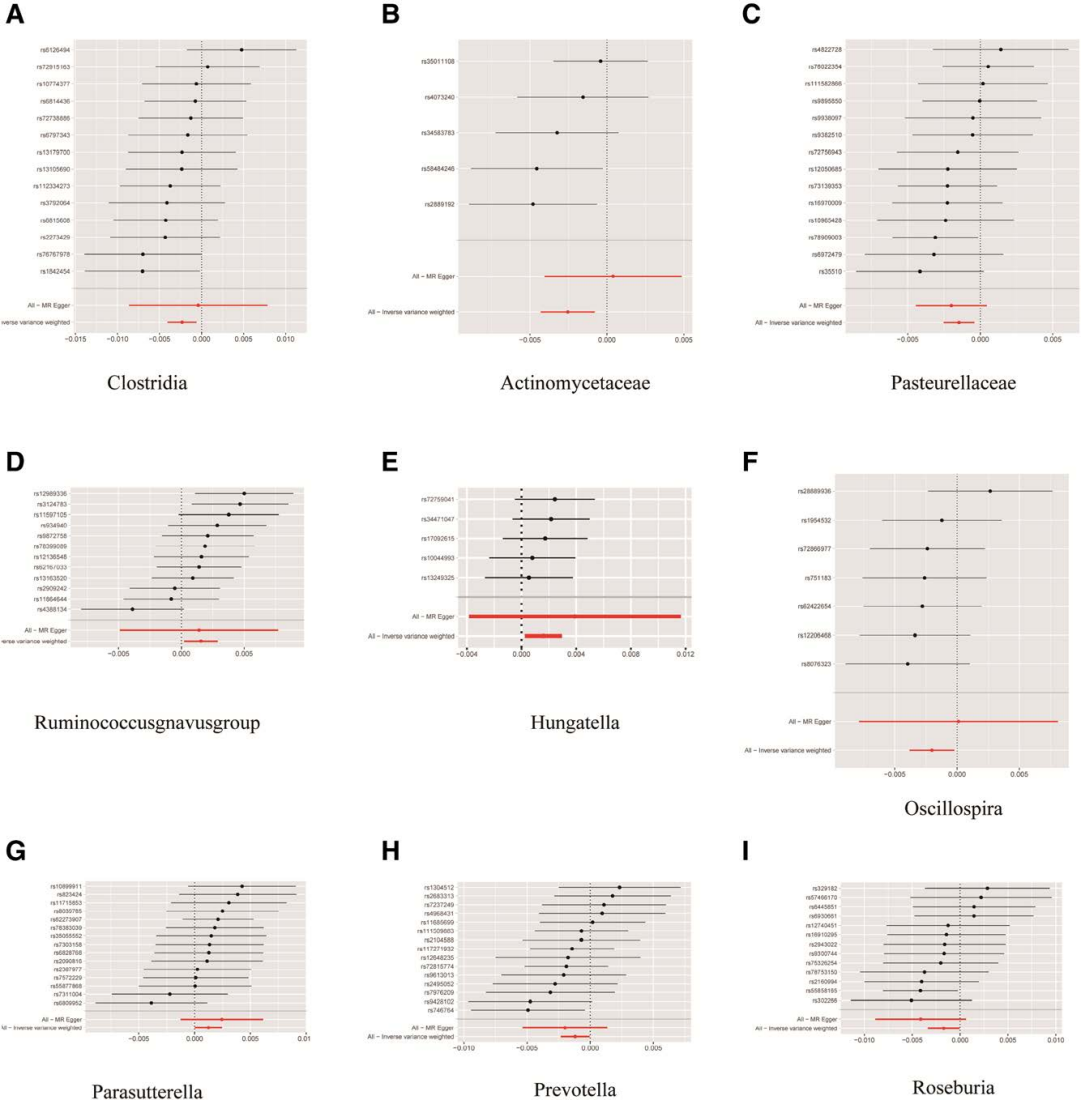
Forest plot depicting the causal association between gut microbiota exposure and IDA. The *x*-axis represents the Mendelian randomization effect size for specific gut microbiota exposure on IDA. The *y*-axis presents the analysis for each of the SNPs. IDA = iron-deficiency anemia, SNPs = single-nucleotide polymorphisms.

**Figure 4.**
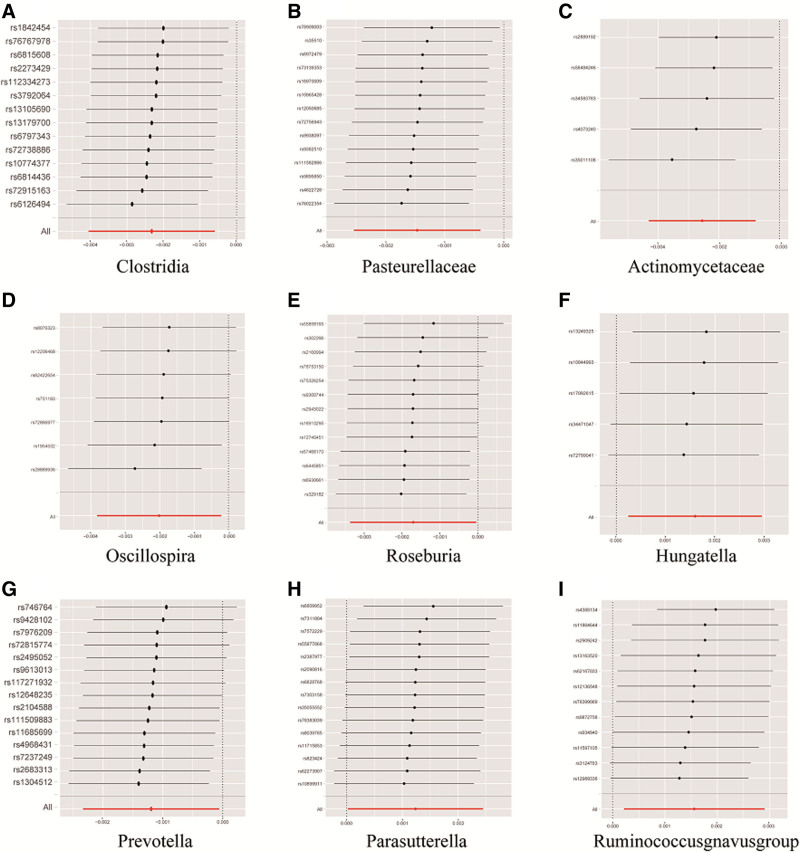
Leave-one-out sensitivity analysis for the effect of gut microbiota exposure on IDA. IDA = iron-deficiency anemia.

In order to assess the stability and reliability of the MR analysis results, additional examinations were conducted, including Cochran IVW *Q* test, MR-Egger intercept analysis, and MR-PRESSO global tests. The Cochran IVW *Q* test indicated no heterogeneity among the IVs (Table 1). Moreover, the MR-Egger regression intercept analysis did not detect significant directional horizontal pleiotropy. Both Cochran *Q* and MR-PRESSO tests showed no heterogeneity (*P* > .05) or outliers in Table 1. Additionally, a visual inspection of the forest plot (Fig. 3) and leave-one-out analysis further confirmed the robustness of the primary findings (Fig. 4).

## 4. Discussion

Upon reviewing relevant databases, we have identified this study as potentially the first to utilize Genome-Wide Association Study (GWAS) summary statistics to explore the genetic associations and potential causal relationships between gut microbiota and IDA. Our findings indicate that 9 gut bacterial species are causally associated with IDA. Among these, Clostridia, Actinomycetaceae, Pasteurellaceae, Oscillospira, Prevotella, and Roseburia are identified as protective factors, whereas the *R gnavus* group, Hungatella, and Parasutterella are recognized as risk factors. Our results suggest that interventions, such as probiotics or gut microbiota modulation, may enhance therapeutic outcomes for IDA, offering insights for the development of future intervention strategies and potential therapeutic targets.

Roseburia is an important genus of symbiotic bacteria in the gut, including various species such as *Roseburia intestinalis*, *R hominis*, *R inulinivorans*, *R faecis*, and *R cecicola*.^[25]^ Current research suggests that dysbiosis (reduced abundance) of Roseburia is associated with a variety of gastrointestinal diseases, including irritable bowel syndrome, ulcerative colitis, and Crohn disease.^[26]^ In these diseases, the abundance of Roseburia is typically lower, leading to a decrease in the production of SCFAs, particularly butyric acid. Additionally, studies have linked Roseburia to metabolic disorders, with the abundance of Roseburia negatively correlated with obesity, type 2 diabetes, and other metabolic diseases.^[25,27]^ Individuals with obesity generally have lower levels of Roseburia, and supplementing Roseburia may help improve metabolic conditions.^[28]^ Neurological studies have shown a significant reduction in Roseburia levels in the intestines of Parkinson disease patients, indicating its importance in protecting the nervous system from invasion.^[29,30]^ Reduction of Roseburia has also been associated with diseases such as atherosclerosis, hypertension, and polycystic ovary syndrome.^[31,32]^ Our study suggests that Roseburia may serve as a protective factor against IDA, highlighting the need for further investigation into the relationship between this gut microbiota and various diseases to aid in the treatment and prevention of these conditions.

Prevotella is a gram-negative anaerobic bacterium that plays a role in the breakdown of protein and carbohydrate foods, contributing to host nutrition absorption and metabolism.^[33]^ Current research indicates that Prevotella exerts significant influence on host metabolism, including reducing visceral fat, improving glucose tolerance, and impacting patient weight.^[34,35]^ *Prevotella nigrescens* and *Porphyromonas gingivalis* are associated with periodontal disease, triggering immune responses and affecting T helper 17 (Th17) cells.^[34]^ Studies have also linked Prevotella to bacterial vaginosis and specific increases in activated cells in the vaginal mucosa.^[36]^ The role of Prevotella in neuroinflammatory diseases and autoimmune inflammatory conditions such as Alzheimer and Parkinson diseases has also been noted.^[37–39]^ Research has further indicated a connection between Prevotella and cognitive decline in middle-aged and elderly individuals.^[40,41]^ Additionally, Prevotella has been closely associated with bacterial vaginosis. For instance, lipopolysaccharides and ammonia produced by *Prevotella bivia* are part of vaginal mucus and can promote the growth of other bacteria associated with vaginal infections, such as *Gardnerella vaginalis*.^[42]^ Therefore, fluctuations in Prevotella abundance may have positive or negative effects on human health. However, in this study, we found that Prevotella plays a protective role in the occurrence of IDA, suggesting the need for further research to elucidate the relationship between Prevotella and IDA.

Oscillospira, widely present in the gastrointestinal tract of animals and humans, is an important component of the gut microbiota.^[43]^ Oscillospira is capable of producing short-chain fatty acids (SCFAs) such as butyrate, which play a crucial role in human health.^[43]^ Numerous studies have shown a strong association between Oscillospira and obesity, obesity-related chronic inflammation, and Parkinson disease, with a significant decrease in Oscillospira abundance observed in these conditions.^[44,45]^ Oscillospira is directly linked to gallstones and chronic constipation, and its abundance may vary in these conditions.^[39,44]^ Due to its significant role in human health and disease, Oscillospira is considered a next-generation probiotic candidate with potential health benefits including weight loss, lipid reduction, and alleviation of metabolic syndrome, indicating significant potential for health applications.^[46]^ Our study further highlights Oscillospira as a protective factor against IDA, underscoring the necessity for additional investigation into the relationship between Oscillospira and IDA, particularly regarding its potential role in the prevention of IDA.

Clostridia are obligate anaerobic, spore-forming, gram-positive rod-shaped bacteria.^[47,48]^ They have the ability to hydrolyze sugars and proteins, inhibit pathogenic bacteria in the gut, and promote the growth of beneficial bacteria such as Bifidobacteria and Lactobacilli.^[49,50]^ Clostridia have been widely used in the prevention and treatment of conditions such as acute and chronic diarrhea resulting from gut dysbiosis, and antibiotic-associated colitis.^[51,52]^ Some actinomycetes in the Actinomycetaceae family have significant applications, including the production of antibiotics, enzyme preparations, and some strains that can cause human diseases.^[53–55]^ The Pasteurellaceae family, which includes genera like Pasteurella, Haemophilus, and Actinobacillus, comprises various important pathogens with significant impacts on human and animal health.^[56–59]^ Research findings indicate that Clostridia, Actinomycetaceae, and Pasteurellaceae serve as protective factors against IDA. This suggests the need to specify which particular bacterial species are beneficial for the treatment of IDA, warranting future randomized controlled trials to clarify the role of these bacteria in improving IDA outcomes.

In addition to the aforementioned bacteria, we also identified *R gnavus* group, Hungatella, and Parasutterella as risk factors for IDA. The *R gnavus* group is associated with various diseases and may serve as a potential biomarker for disease diagnosis. Current studies suggest that Hungatella can lead to sepsis in humans, causing severe human diseases. Parasutterella, a bacterial genus primarily found in the gut microbiota of humans and mice, is considered a core member of these gut microbiota communities.^[60,61]^ Research indicates that *Parasutterella excrementihominis* can reduce blood glucose levels in high-fat diet-induced obesity and insulin resistance models in mice, as well as improve inflammation in mouse adipose tissue and alleviate hepatic steatosis.^[60,62,63]^

However, there are some limitations in this study. Firstly, the gut microbiome can be influenced by various factors such as diet, medication, demographic factors, among others. Most of these factors exhibit inter-individual variability and low heritability (representing the variance explained by genetics), which reduces the statistical power and robustness of the results. Secondly, although the majority of individuals in the gut microbiome GWAS meta-analysis are of European descent, there is still a possibility of interference from a small number of participants from other ethnicities, potentially leading to biases and affecting the generalizability of the results. Additionally, in order to achieve more comprehensive results and to conduct horizontal pleiotropy testing and sensitivity analysis, the selected genetic IVs did not reach the conventional GWAS significance threshold (*P* < 5 × 10^−8^), which could increase the likelihood of false positives.

## 5. Conclusion

In summary, this study provides evidence of genetic correlations among 9 gut bacteria, identifying Clostridia, Actinomycetaceae, Pasteurellaceae, Oscillospira, Prevotella, and Roseburia as protective factors. Future clinical studies are needed to further clarify whether the aforementioned bacteria are beneficial for the treatment of IDA.

## Acknowledgments

The authors express their gratitude to the participants and investigators involved in all GWAS included in this research. The authors also acknowledge the MiBioGen Consortium and the UK Biobank for making the summary data publicly accessible.

## Author contributions

**Conceptualization:** Zhaoyun Liu, Rong Fu.

**Funding acquisition:** Hai-Ping Lai.

**Methodology:** Rong Fu.

**Software:** Hai-Ping Lai, Rong Fu.

**Writing – original draft:** Wenhui Lei.

**Writing – review & editing:** Wenhui Lei, Zhaoyun Liu, Hai-Ping Lai, Rong Fu.
